# Pulsed Radiofrequency Neuromodulation of the Greater Occipital Nerve for the Treatment of Headache Disorders in Adults: A Systematic Review

**DOI:** 10.1080/24740527.2024.2355571

**Published:** 2024-05-15

**Authors:** Kyle De Oliveira, Nina Dhondt, Marina Englesakis, Akash Goel, Yasmine Hoydonckx

**Affiliations:** a Department of Anesthesia and Pain Management, Sunnybrook Health Sciences Centre, Toronto, Ontario, Canada; b Department of Pain Medicine, Multidisciplinary Pain Center, VITAZ, Sint-Niklaas, Belgium; c The Institute of Education Research, Library & Information Services, University Health Network, Toronto, Ontario, Canada; d Department of Anesthesia and Pain Management, St Michael's Hospital, Unity Health, Toronto, Ontario, Canada; e Department of Anesthesia and Pain Medicine, Toronto Western Hospital, Toronto, Ontario, Canada

**Keywords:** Pulsed radiofrequency neuromodulation, headache, migraine, hemicrania, cephalgia, cephalalgia, occipital, occipital neuralgia

## Abstract

**Background:**

Pulsed radiofrequency neuromodulation (PRFN) of greater occipital nerve (GON) is considered in patients with headaches failing to achieve sustained analgesic benefit from nerve blocks with local anesthetic and steroids. However, the evidence supporting this practice is unclear.

**Aims:**

This narrative systematic review aims to explore the effectiveness and safety of GON PRFN on headaches.

**Methods:**

Databases were searched for studies, published up to February 1, 2024, investigating PRFN of GON for adults with headaches. Abstracts and posters were excluded. Primary outcome was change in headache intensity. Secondary outcomes included effect on monthly headache frequency (MHF), mental and physical health, mood, sleep, analgesic consumption, and side-effects. Two reviewers screened and extracted data.

**Results:**

Twenty-two papers (2 randomized controlled trials (RCT), 11 cohort, and 9 case reports/series) including 608 patients were identified. Considerable heterogeneity in terms of study design, headache diagnosis, PRF target and settings, and image-guidance was noted. PRFN settings varied (38–42°C, 40–60 V, and 150–400 Ohms). Studies demonstrated PRFN to provide significant analgesia and reduction of MHF in chronic migraine (CM) from 3 to 6 months; and significant pain relief for ON from six to ten months. Mild adverse effects were reported in 3.1% of cohort. A minority of studies reported on secondary outcomes. The quality of the evidence was low.

**Conclusions:**

Low-quality evidence indicates an analgesic benefit from PRFN of GON for ON and CM, but its role for other headache types needs more investigation. Optimal PRFN target and settings remain unclear. High-quality RCTs are required to further explore the role of this intervention. PROSPERO ID CRD42022363234.

## RÉSUMÉ

**Contexte**: La neuromodulation par radiofréquence pulsée (NRFP) du nerf grand occipital (NGO) est envisagée chez les patients souffrant de céphalées qui ne parviennent pas à obtenir un bénéfice analgésique durable à partir des blocages nerveux à l’aide d’un anesthésique local et de stéroïdes. Cependant, les données probantes à l'appui de cette pratique ne sont pas claires.

**Objectifs**: Cette revue systématique narrative vise à explorer l'efficacité et la sécurité de la NRFP du NGO sur les maux de téte.

**Méthodes**: Des bases de données ont été consultées pour trouver des études, publiées jusqu'au 1er février 2024, portant sur la NRFP du NGO chez des adultes souffrant de céphalées. Les résumés et les affiches ont été exclus. Le critére principal était le changement dans l'intensité des maux de téte. Les critéres secondaires comprenaient l'effet sur la fréquence mensuelle des céphalées, la santé mentale et physique, l'humeur, le sommeil, la consommation d'analgésiques et les effets secondaires. Deux examinateurs ont évalué et extrait les données.

**Résultats**: Vingt-deux articles (2 essais contrôlés randomisés, 11 cohortes et 9 rapports de cas/séries) portant sur 608 patients ont été recensés. Une hétérogénéité considérable a été observée en termes de devis de l'étude, de diagnostic des céphalées, de la cible et des paramétres de la FRP et de l'orientation de l'image. Les réglages de la NRFP variaient (38-42°C, 40-60 V, et 150-400 Ohms). Les études ont démontré que la NRFP procurait une analgésie significative et réduisait la fréquence des céphalées dans la migraine chronique de trois à six mois, et un soulagement significatif de la douleur pour la névralgie occipitale pendant six à dix mois. Des effets indésirables légers ont été signalés dans 3,1 % des participants de la cohorte. Une minorité déétudes ont fait état de résultats secondaires. La qualité des données probantes était faible.

**Conclusions**: Les données probantes de faible qualité indiquent un bénéfice analgésique de la NRFP du NGO pour la névralgie occipitale et la migraine chronique, mais son rôle pour d'autres types de céphalées doit être davantage étudié. La cible et les paramétres optimaux de la NRFP restent floues. Des essais contrôlés randomisés de haute qualité sont nécessaires pour explorer davantage le rôle de cette intervention.

## Introduction

Headache disorders are a common affliction worldwide affecting approximately 15% of the world’s population.^1^ There is a wide spectrum of headache disorders that are largely classified as either primary or secondary according to the International Classification of Headache Disorder–3rd Edition (ICHD-3).^2^ Primary headache disorders include migraine, tension-type, and trigeminal autonomic cephalagias (TACs) that can all differ widely in their respective headache burden, pathophysiology, and management.^3,4^ Occipital neuralgia (ON), another notable headache diagnosis, is classified as a cranial neuropathy under part three of ICHD-3. Although, the medical management of these headache disorders has evolved and progressed substantially, it is estimated that more than 5% of patients remains refractory to treatments.^5^

The trigeminocervical complex (TCC) has been proposed to play a major role in the central sensitization and pathogenesis of headaches.^6^ The TCC is located within the brainstem and is the primary site where nociceptive, small-diameter cervical, dural, muscle and trigeminal afferents converge.^6^ The greater occipital nerve (GON) carries with it the cervical, supratentorial dura (visceral) and muscle afferents that play a crucial role in the central sensitization and hyperexcitability of the entire converging neuronal network that forms the TCC.^6^ As a result, in cases of refractory headache or craniofacial pain, prevailing theories support the notion that blocking or modulation the sensory inputs from the GON to the TCC may be helpful. The GON arises from the dorsal ramus of the C2 spinal nerve (dorsal root ganglion, ‘DRG’) and travels cranially in the suboccipital area through the semispinalis capitis muscle (SSCM) and the obliquus capitis inferior muscle (OCIM) before it arrives at the occiput and continues its course cranially over the skull along the occipital artery up to the vertex of the skull.^7^

Landmark-guided (LMG) or ultrasound-guided (USG) GON blocks (GONB) with local anesthetics (LA) and/or steroids or botulinum toxin have proven their efficacy in reducing headache intensity and headache frequency for limited time.^8–10^ Three targets for injecting or blocking the GON have been proposed: 1) close to the origin of the nerve at the level of C2 DRG (“DRG approach”); 2) along the proximal part of the GON while it is running suboccipital in the plane between the SSCM and OCIM (the “proximal approach”); or along the distal part of the GON at the level of the superior nucheal line (the “distal approach” approach). Image-guidance is required to perform the DRG-approach or proximal approach, but the distal approach is frequently performed under LMG. In terms of efficacy between targets, the literature on GON injections suggests similar results when comparing between the described distal and proximal approaches.^11–13^ Adverse effects have been reported as minimal across studies regardless of approach.^11–14^

Although beneficial, the literature on GONB with injectates as LA and steroid only suggests short-term efficacy from GONB lasting weeks to few months in the treatment of different headache disorders.^12,14,15^ With hopes of extending the therapeutic benefit observed, a newer technique called pulsed radiofrequency neuromodulation (PRFN) has been proposed and investigated. This technique involves treating the nerves with radiofrequency waves generated by electric currents to prevent conduction of nociceptive signals. The mechanism of action is not yet fully understood but is thought to involve an alteration of the synaptic transmission, which creates a neuromodulatory-type effect.^16^ Previous studies have identified PRFN as causing extended c-Fos activation in rats, potentially due to selective long-term depression in C-fiber–mediated spinal sensitization.^17^ Other mechanisms proposed are possible microscopic thermal damage to thinly myelinated A-delta and unmyelinated C-fibers and reduction of proinflammatory cytokines.^18,19^ PRFN of a wide arrange of nerves and ganglions in the body, has proven to provide relief for several months to over a year, in the treatment of complex chronic pain states including axial pain, trigeminal neuralgia and facial pain, inguinal neuralgia, and other neuropathic pain states.^20–27^

The therapeutic role of PRFN of the GON in the treatment of headache disorders has not been completely elucidated. Therefore, the objective of this systematic review was to evaluate the effectiveness and overall safety of PRFN of the GON in the treatment of headache disorders.

## Methods

### Registration

This systematic review (SR) was conducted according to the recommendations of the Cochrane Collaboration and was reported as per the Preferred Reporting Items for Systematic Reviews and Meta-Analysis (PRISMA) guidelines. The protocol of this SR was registered with PROSPERO (ID CRD 42022363234).

### Data Sources and Search Strategy

We conducted a comprehensive search of the literature with the assistance of a medical information specialist from inception to November 1, 2022. An updated search was conducted over the same databases on February 1 2024.The following databases were searched: Embase Classic/Embase, 1947 onward; MEDLINE, 1946 onward; MEDLINE ePubs, In-Process and Other Non-Indexed Citations; Cochrane Database of Systematic Reviews, 2005 onward; and Cochrane Central Register of Controlled Trials, 1991 onward, (all using the Ovid Platform); and PubMed-NOT-MEDLINE (NLM NIH). We also searched Web of Science Core Collection, 1900 onward (Clarivate Analytics) and Scopus, 1960 onward (Elsevier). Clinical trial registries, ClinicalTrials.Gov (NIH) and WHO ICTRP (International Clinical Trials Registry Platform), were searched to identify trials. We restricted our search to human subjects. No language restrictions were implemented. For Embase, MEDLINE, Cochrane CENTRAL, and Scopus, both controlled vocabulary terms (Embase-Emtree; MEDLINE-MeSH) and text word searching were conducted. Both our initial and most recent Ovid MEDLINE detailed search strategies are provided in Appendices 1a, 1b, 2a, and 2b.

### Inclusion/Exclusion Criteria

The studies were screened for eligibility based on title, abstract, and full text for the following criteria:

#### Population

This review included studies on human subjects of ≥ 18 years of age diagnosed with a headache disorder and/or ON according to the International Classification of Headache Disorder-3rd Edition (ICHD-3).^2^ No restrictions were put in terms of duration and frequency of headache. Studies that investigated effects of PRFN on a mixed population of pain patients for which relevant data could not be extracted separately were excluded.

#### Intervention

The intervention was defined as PRFN of the GON. Any approach (distal or proximal approach on the GON or C2 DRG) was accepted. There was no limit on the duration of the treatment or number of treatments.

#### Comparator

Comparators included no treatment, placebo treatment or conventional medical management, which could include pharmacological, physical, psychological, and/or interventional therapies.

#### Outcome

The primary outcome of interest was the effect on pain (headache) intensity, as measured by the numeric rating scale (NRS) or visual analogue scale (VAS). Secondary outcomes of interest included: 1) headache frequency, 2) mental and physical outcome, 3) mood, 4) sleep, 5) analgesic consumption, 6) quality of life, 7) patient satisfaction.

#### Study Designs

The scope of studies included randomized controlled trials, systematic reviews, observational or cohort studies, case-control studies, and case series/reports.

### Study Selection Process

All citations were independently screened on title and abstract for eligibility by two reviewers (K.D. and N.D.) as per the inclusion criteria. Covidence® was used as a systematic review management tool. Papers of interest were then full text screened. Of the selected papers, data was independently extracted by two reviewers (K. D. and N.D.). Any apparent conflicts were resolved through discussion with senior author (Y.H.).

### Data Extraction

The reference data, populations, and outcomes were extracted from the articles into pre-specified tables on a standardized data collection form in Microsoft Word that was pilot tested before use. Extracted data for each study included: general characteristics (first author, publication year, number of patients, and study design), patient characteristics (age, gender), clinical information (diagnosis, duration, baseline headache intensity and headache frequency), details of intervention (approach – C2 DRG/distal/proximal, PRFN settings, type of image guidance, duration of therapy) and comparator (dose, duration, regimen), data on primary and secondary outcomes of interest, follow-up time points, and adverse effects. For continuous data, means (or medians) and standard deviations (or interquartile ranges or ranges) were extracted.

### Assessment of Quality as Risk of Bias

Two review authors (K.D. and N.D.) independently assessed the risk of bias for randomized controlled trials (RCTs) using the Cochrane Risk of Bias tool 2.0.^28^ Any disagreement was resolved through discussion with senior author (Y.H.). The Robins-I was used to assess the risk of bias in observational studies.^29^ For case reports/case series, the Quality Appraisal Tool for Case Series was used.^30^

### Data Synthesis and Analysis

We narratively synthesized the characteristics of all studies that met inclusion criteria. Study characteristics and study outcomes were summarized. No meta-analysis was performed due to the heterogeneity of data and small sample sizes reported.

## Results

### Search Results

**Figure 1.**
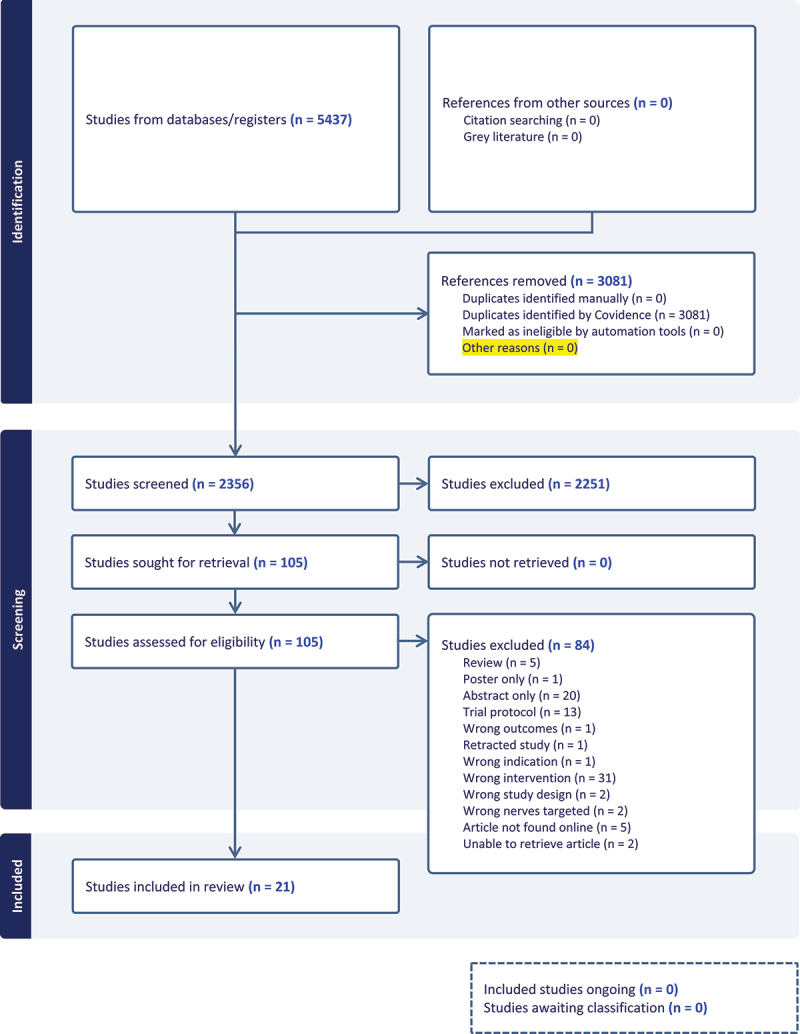
PRISMA Flow Diagram.

**Figure 2.**
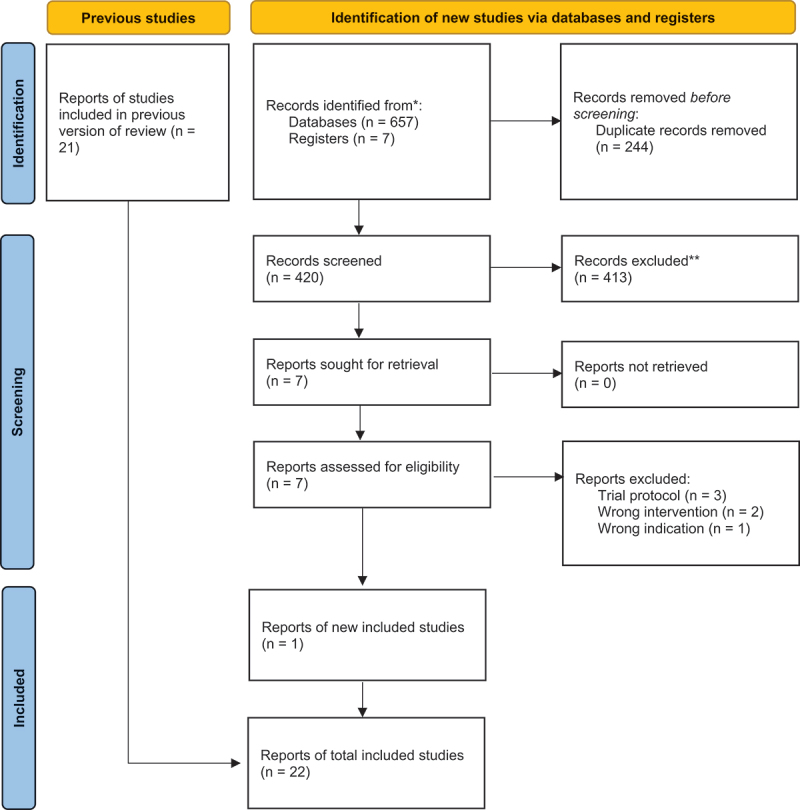
PRISMA Flow Diagram for Updated Systematic Review.

The initial search strategy retrieved a total of 2356 studies, of which 2251 studies were excluded at the screening stage. Full texts of 105 articles were assessed for eligibility, of which 21 studies deemed to meet all eligibility criteria for inclusion in this review. Our updated search retrieved an additional 664 studies, of which 657 studies were excluded at the screening stage. Full texts of 7 articles were assessed for eligibility, of which 1 study was deemed to meet all eligibility criteria for inclusion in this review. Finally, two RCTs,^31,32^ four prospective cohort studies,^33–36^ seven retrospective cohort studies,^37–43^ two case series,^44,45^ and 7 case reports were included.^46–52^ A summary of the original search strategy is described in Figure 1 (PRISMA Original Search) and the updated search in Figure 2 (PRISMA Updated Search).

*Consider, if feasible to do so, reporting the number of records identified from each database or register searched (rather than the total number across all databases/registers). **If automation tools were used, indicate how many records were excluded by a human and how many were excluded by automation tools. *From*: Page MJ, McKenzie JE, Bossuyt PM, Boutron I, Hoffmann TC, Mulrow CD, et al. The PRISMA 2020 statement: an updated guideline for reporting systematic reviews. BMJ 2021;372:n71. doi: http://doi.org/10.1136/bmj.n71. For more information, visit: http://www.prisma-statement.org/.

### Risk of Bias for Included Studies

The results for assessment of the risk of bias for the included RCTs are summarized in Table 3. The overall risk of bias was deemed to be of “some concerns” for both included RCTs.^31,32^ The risk of bias assessment of the included cohort studies revealed five studies were deemed to be at moderate risk of bias,^34–38,40-43^ three studies were found to be at serious risk,^39-41,42^ and one critical (Table 4).^33^ The risk of bias of the case series was moderate (Table 5).^44,45^

### Study Characteristics

Demographic and clinical characteristics of all PRFN treated patients reported in the 22 included studies are summarized in Table 1. The use of PRFN for the management of a primary headache disorder (migraine disorder, tension type, TAC) was reported in six cohort studies^43–48^ and three case reports/series.^49–51^ The use of PRFN for the management of a secondary headache disorder (cervicogenic headache or headache caused by atlantoaxial instability) was reported in seven studies: one RCT,^32^ four cohort studies^35,40–42^ and two case reports.^49,52^ PRFN was used in the management of ON in a total of six studies: one RCT,^31^ four cohort studies^34,36,37,39^ and one case report.^51^

**Table 1.** Characteristics of studies included in the systematic review: participants, interventions, comparators and outcome measures.

1st author, year, number of patients, type of study	Participants: 1. Age 2. Gender (M/F) 3. Diagnosis 4. Duration of pain 5. Baseline pain score 6. HA frequency	Details of PRF: 1. Settings 2. Location: DA/PA/DRG 3. Type of image-guidance 4. Duration 5. Number of PRF treatments	Diagnostic block prior to PRF	Comparator (settings, dose, duration)	
**RANDOMIZED CONTROLLED TRIALS**					
Cohen 2015^31^ n = 81 P	1. Mean age (SD): 41.67 (±10.72) yrs 2. M/F: 22/20 3. ON (n = 42), migraine (n = 25) 4. 6.44 (±7.39) yrs 5. Mean NRS occipital:5.77 ± 1.34 overall headache: 44 ± 1.24 6. Mean (SD) # weekly HA days: CM 3.64 (±2.10); ON 3.18 (±2.19)	1. 42°C, 40–60 V, 2 Hz, 150–400 Ω, 20 ms PW 2. DA 3. LMB 4. 3 × 120 s 5. one	Yes (LA + steroid)	GONB (LA + steroid) + sham-PRF	
Gabrhelík 2011^32^ n = 30 P Pilot trial	1. Median age (IQR): 43.60 (28–65) yrs 2. M/F: 6/9 3. CEH 4. NR 5. Mean (SD) NRS 5.90 (± 1.2) 6. NR	1. 42°C, 45 V 2. DA 3. LMB 4. 2 × 120 s 5. one	Yes (LA only)	GONB (steroid)	
**NON-RANDOMIZED STUDIES**					
Alkaabi 2020^33^ n = 30 P	1. Mean age (SD): 30.03 ± 6.64 yrs 2. M/F: 6/24 3. Migraine (60%) 4. >10 yrs: 13/30; 5–9 yrs: 14/30; 1–4 yrs: 10/30 5. NR 6. NR	1. 42°C, 42 V, 2 Hz, 100 Ω, 230 mA, 150–500 W 2. DA 3. LMG 4. 3 × 3 min. 5. one	NR	N/A	
Batistaki 2021^34^ n = 57 P	1. CM: 51.79 ± 13.67 yrs; CH: 57.66 ± 12.52 yrs; ON/TTH:55.9 ± 12.33 yrs 2. NR 3. *CM (n = 38) *CH (n = 9) *ON/TTH (n = 10) 4. Median (IQR)yrs: CM: 20 (10–34) yrs CH:15 (5–20)yrs ON/TTH:5 (3–36) yrs 5. NRS CM: 8.45 ± 1.28; CH: 8.55 ± 0.88; ON/TTH: 8 ± 1.41 6. Median HA days/month: CM: 14.5 (8–25) CH:15 (15–30) ON/TTH:16.5 (15–30)	1. 42°C, 40–60 V, 2 Hz, 150–400 Ω, 2. DA 3. LMG 4. 6 min 5. one	Yes (NR)	N/A	
Choi 2012^37^ n = 10 R	1. Median age (IQR): 52 (34–70) yrs 2. M/F: 3/7 3. ON 4. NR 5. mean (SD) VAS 6.9 (NR) 6. range: daily – monthly	1. 42°C, 240 pulses 2. DA 3.XRG 4. NR 5. one	Yes (LA + DXM)	N/A	
Guner 2022^38^ n = 25 R	1. Median age (IQR): 47 (35–62 years) yrs 2. M/F: 8/17 3. CM without aura 4. NR 5. VAS median (IQR):9 (8–10) 6. median (IQR) HA frequency: 10 (8–12) episodes median (IQR) duration: 48 h (24–72)	1. 42°C, 45 V, 5 Hz, PW 5 ms 2. PA 3. USG 4. 360 s 5. one	Yes (NR)	N/A	
Huang 2012^39^ n = 102 R	1. Mean age (SD): 51.2 ± 14.5 yrs 2. M/F: 27/75 3. ON or CM 4. Mean (SD): 6.9 ± 8.6 yrs 5. NR 6. NR	1. 42°C, 40–60 V,2 Hz, 150–500 Ω,PW 20 ms 2. DA 3. LMB 4. 120s 5. one (n = 41) or ≥ two (n = 61)	Yes (LA + MP)	N/A	
Karaoglan 2022;^43^ n = 27 R	1. Mean age (SD): 35.70 ± 4.45 yrs 2. M/F: 4/23 3. CM or EM 4. Mean (SD): 8.37 ± 3.58 yrs 5. Mean (SD) Highest VAS score: 8.89 ± 0.75 6. Mean (SD) Migraine attacks per month: 12.04 ± 2.83	1. 42°C, 45 V, 5 Hz, PW 5 mm 2. DA 3. LMB 4. 360s 5. one	Diagnostic block (bupi)	N/A	
Lee 2020^40^ n = 45 R	1. Median age (IQR) 63 (53–72) yrs 2. M/F: 21/24 3. CEH 4. median (IQR) 12 (5–38) months 5. Median (IQR) NRS (IQR) 6 (5–8) 6. NR	1. 42°C 2. DRG 3. XRG 4. 3x 90 s 5. one	Yes (NR)	N/A	
Li 2020^35^ n = 20 P	1. Mean (SD) age: 46.5 ± 8.5 yrs 2. NR 3. Chronic HA (n = 16), CEH (n = 2), TTH (n = 1), CCH (n = 1) 4. > 5 yrs 5. mean (SD) VAS: 7.1 (NR) 6. NR	1. 42°C, 2 Hz, 65 V, PW 20 ms 2. DRG 3. CTG 4. 900 s 5. one	Yes (NR)	N/A	
Li 2019^41^ n = 139 R	1. mean age (SD) 47.5 ± 12.4 yrs 2. M/F: 91/48 3. CEH 4. 50.4 ± 37.6 (3–180) m 5. median (IQR) Izbicki pain score: PRF+ESI 78.5 (NR), ESI 72.5 (NR) (p = 7.574) 6. constant HA: 86/139: intermittent daily: 38/139: intermittent weekly: 15/139	1. 42°C, 60 Hz 2. DRG 3. NR 4. 5 min 5. one	NR	C-ESI (LA + steroid)	
Vanelderen 2010^36^ n = 19 P	1. M: median (IQR): 57.6 (43–72) yrs; F: median (IQR): 53.3 (22–77) yrs 2. M/F: 5/14 3. ON 4. NR 5. Mean (SD) VAS 7.5 ± 0.4 6. NR	1. 42°C, 2 Hz, 45 V, PW 20 ms 2. DA 3. LMG 4. 240 s 5. one	Yes (LA only)	N/A	
Van Zundert 2003^42^ n = 4 R	1. 29, 53, 40, 60 yrs 2. M/F: 1/3 3. CEH 4. 2 to 3 yrs 5. NR 6. NR	1. 42°C max, 45 V, PW 20 ms 2. DRG 3. NR 4. 120 s 5. one	Yes (LA only)	N/A	
**CASE SERIES/CASE REPORTS**					
Belgrado 2021^44^ n = 4 R	1. 55, 55, 52, 82 yrs 2. M:4 3. CCH (n = 1), ECH (n = 3) 4. Median (IQR) 25 (3–36) yrs 5. NR 6. 6 (2–8) attacks/d	1. 42°C, 2 Hz, PW 20 ms 2. DA 3. NR 4. 2 × 180 s 5. one (n = 2), two (n = 1), three (n = 1)	Yes (NR)	N/A	
Fadayomi 2019^46^ n = 1 R	1. 32 yrs 2. M 3. CCH 4. 3 yrs 5. NR 6. 6–10 attacks/d	1. 42°C 2. DRG 3. USG 4. 90 s 5. one	Yes (LA + steroid)	N/A	
Gonzales 2020^47^ n = 1 R	1. 75 yrs 2. F 3. SUNCT 4. Several years 5. NR 6. 2–5 attacks/d, lasting for 30s to 3 min	1. 42°C 2. DA 3. NR 4. 2 × 120 s 5. two	Yes (LA + steroid)	N/A	
Kwak 2018^48^ n = 2 R	1. 33 y, 34 yrs 2. 1/1 3. CM 4. 15 yrs, 14 yrs 5. NRS 8/10 and 7/10 6. Daily headache, 12–48 h duration of attacks	1. 42°C, 5 Hz, 45 V, PW 5 ms 2. PA 3. USG 4. 360 s	Yes (LA + steroid)	N/A	
Lee 2015^49^ n = 1 R	1. 74 yrs 2. F 3. occipital headache 4. 2–3 years 5. 6/10 neck pain; 10/10 occipital pain with neck movement 6. continuous	1. 42°C 2. DRG 3. NR 4. 3 × 120 s 5. one	Yes (LA + steroid)	N/A	
Li 2018^50^ n = 1 R	1. 34 yrs 2. F 3. CM 4. > 10 yrs 5. VAS 8/10 6. Daily	1. 42°C, 45 V, 2 Hz, PW 20 ms 2. DRG 3. USG + CTG 4. 900 s 5. one	Yes (LA only)	N/A	
VanderHoek 2013^51^ n = 1 R	1. 32 yrs 2. M 3. ON 4. NR 5. NR 6. NR	1. 38–42°C, 2 Hz, PW 20 ms 2. DA 3. USG 4. 2 × 120 s 5. one	NR	N/A	
Voloshin 2021^45^ n = 6 R	1. 33–57 yrs 2. M/F: 0/6 3. CM 4. 16–44 yrs 5. Mean (SD) NRS 7 ± 1 6. Mean (SD) 22 ± 5 d per month	1. 42°C, 65 V, 5 Hz, PW 5 ms 2. PA 3. USG 4. 360 s 5. one	No	N/A	
Zhang 2011^52^ n = 2 R	1. 40 yrs, 66 yrs 2. M/F: 1/1 3. CEH 4. 5 yrs and 1 yrs 5. NRS 5 and 4 6. NR	1. 42°C 2. DRG C2 3. XRG 4. 4 min 5. one	Yes (LA only)	N/A	

AAB: atlanto-axial block; AB: beta-adrenoblockers; AE: antiepileptic drugs; AD: antidepressants; BM: betamethasone; C: Celsius; CCH: Chronic Cluster Headache; CESI: Cervical epidural steroid injection; CGRP: calcitonin gene-related peptide receptor; CEH: Cervicogenic headache; CM: Chronic Migraine; CMBB: Cervical medial branch block; CTG: CT-guided; DA: distal approach; DXM: dexamethasone; ECH: Episodic Cluster Headache; C-ESI: Cervical epidural Steroid Injection; F: female; GONB: Greater Occipital Nerve Block; HA: headache; LA: local anaesthetic; Lido: lidocaine; LMB: landmark-based; LONB: lesser Occipital Nerve Block; M: male; Mepi: mepivacaine; MP: methylprednisolone; MR: muscle relaxant; N/A: not applicable; NR: not reported; NS: not specified; NSAID: nonsteroidal anti-inflammatory drug; ON: Occipital Neuralgia; PA: proximal approach; P: prospective study; PRF: pulsed radiofrequency neuromodulation; PPF: pterygopalatine fossa; pulse width: PW; RA: Rheumatoid Arthritis; RCT: randomized controlled trial; NaCl: saline; SD: sartan drugs; SGB: Stellate Ganglion Block; SIHH: Spontaneous intracranial hypotension headache; SNRI; serotonin and norepinephrine reuptake inhibitor; SSRI; selective serotonin reuptake inhibitor; SUNCT: Short-lasting Unilateral Neuralgiform headache attacks with Conjunctival injection and Tearing; TCA: tricyclic antidepressants; TENS: transcutaneous electric neurostimulation; TONB: Third Occipital Nerve block; TC: triamcinolone; TTH: Tension-type headache; USG: ultrasound-guided; XRG: fluoroscopy-guided; V: volt; W: Watt; Yrs: years; #: number

### Patient Characteristics

A total of 608 patients were included in this review, age ranging from 22 to 82 years. Gender was not included for 77 of the 608 patients.^34,35^ Of the remaining 531 patients, 52% were female. Two hundred and twenty-three (36.7%) subjects had a diagnosis of ON, 222 (36.5%) were afflicted with cervicogenic headaches (CEH), 118 (19.4%) suffered with chronic migraine (CM), and 15 (2.5%) experienced cluster headaches (CH). One patient each (0.16%) remained for short-lasting unilateral neuralgiform headache attacks with conjunctival injection and tearing (SUNCT), tension type headache disorder (TTH), headache caused by atlantoaxial instability. Baseline headache intensity was moderate to severe, ranging from 4/10 to 9/10 on a Numerical Rating Scale (NRS).

### PRFN Targets

Three different interventional targets were found: PRFN was applied at the level of C2 DRG in eight studies,^35,40–42,46,49,50,52^ as a “distal GON approach” in 11 studies,^31–34,36,37,39,43,44,47,51^ in a “proximal GON approach” in three studies.^38,45,48^

### PRFN Settings

Treatment parameters for PRFN consisted of one,^31–38,40–43,45,46,48–52^ two,^39,47^ or three^44^ cycles on a temperature of 42°C for a duration of 90^40^ or 120^39,42,47,49,51^ to 360^34,38,43,45,48^ seconds at 40–60 volts.^31,34,36,38,39,42,43,48,50^ Two outliers reported one cycle of 900 seconds.^35,50^ Pulse widths were indicated as either five^38,43,45,48^ or twenty milliseconds.^31,35,36,39,42,44,50,51^

### PRFN Single Cycle

Nineteen out of 22 included studies utilized a single PRFN treatment cycle: all studies on use of PRFN at C2 DRG level^35,40–42,46,49,50,52^ and at “proximal GON approach,”^38,45,48^ and 8 out of 11 studies exploring PRFN at the “distal GON approach.”^31–34,36,37,43,51^

### Image Guidance

Five papers performed PRFN utilizing USG,^38,45,46,48,51^ seven studies reported LMG technique,^31–34,36,39,43^ three studies used X-ray guidance (XRG),^37,40,52^ one study used CT-guidance (CTG)^35^ and one study combined CTG and USG.^50^ The remaining five studies did not comment on use of image guidance.^41,42,44,47,49^

### Duration of Follow-up

Follow-up times ranged from one week,^35,44^ two weeks,^31,46,48^ one month,^33,34,36,38,40,45^ two months,^42^ three months,^32,39^ six months,^37^ one year^50^, to two years after treatment.^41^ In four studies, follow-up timepoints were not specified.^47,49,51,52^

### Duration of Analgesic Benefit

Duration of analgesic benefit from PRFN was reported in a total of 21 studies^31–50,52^ and ranged from 3 months,^34,38,39,48^ 4 months,^45^ 6 months^31,35,39,40,43,44,49,50,52^, 8 months^36,41,46^, 9 months^32^, 12 months^33,42,47^, 15 months^39^, 18 months to 2 years.^37,39^

### Secondary Health Outcomes

A total of eight studies reported on MHF.^31,34,38,43–45,47,48^ Eight studies reported on analgesic consumption.^32,34,36,37,42,43,45,47^ Four studies reported on QOL measures.^31,36,38,41^ Two studies reported on the impact of PRFN treatment on quality of sleep.^38,41^

### PRF Effects on Headache Disorders Stratified by PRF Targets

#### C2 Drg

We found eight studies (four cohort studies,^35,40–42^ four case reports^46,49,50,52^) examining the effects of PRFN treatment to the DRG of C2 for CEH,^35,40–42,52^ CH,^46^ CM,^50^ and headache caused by atlantoaxial instability.^49^ Two studies used an XRG technique,^40,52^ one study used CTG,^35^ one study used USG,^46^ and one case report used a combined approach with CTG and USG.^50^ Three studies did not specify any use of image guidance technique.^41,42,49^

#### Summary of Results with C2 DRG Approach

A total of 213 patients across eight studies were analyzed that utilized the C2 DRG approach to PRFN in the treatment of headaches.^35,40–42,46,49,50,52^ For CEH, two studies^35,41^ and one case report^52^ (n = 161) out of five studies were deemed to demonstrate significant pain relief up to 6 months^35,52^ or a median duration of effect of 8 months.^41^ We found one case report each for the indication of chronic CH, CM, and AAS, with patients having significant pain relief from 6 months up to 24 months.

#### CEH

Five studies (four cohort studies,^35,40–42^ one case report^52^) investigated the effect of PRFN on C2 DRG for patients suffering from CEH. (Table 2)

**Table 2.** Characteristics of studies included in the systematic review: follow-up times, results and adverse effects.

1st author, year, number of patients, type of study	Outcomes assessed	Follow-up time points	Results: PRF (P) vs Comparator (C)	Adverse Effects	Study outcome
**RANDOMIZED CONTROLLED STUDIES**					
Cohen 2015;^31^ n = 81; P	Primary: *occipital pain (NRS) Secondary: *Worst occipital pain (NRS) *Average and worst overall HA (NRS) *HA frequency *rescue medication *GPE *HIT-6	2 w, 6 w, 3 m, 6 m	1. *Mean (SD) average occipital pain: 2 w: 2.495 ± 2.094 (P) vs 3.691 ± 2.221 (C) (p < 0.001), 6 w: 3.068 ± 2.431 (P) vs 3.738 ± 2.415 (C) (p < 0.008) 3 m: 3.791 ± 2.573 (P) vs 4.441 ± 2.237 (C) (p < 0.020) 6 m: 4.312 ± 2.232 (P) vs 4.765 ± 1.998 (C) (p < 0.017) *Mean (SD) Worst occipital pain: 2 w: 4.726 ± 2.528 (P) vs 5.846 ± 2.618 (C) (p < 0.033) 3 m: 5.850 ± 3.292 (P) vs 7.149 ± 2.967 (C) (p < 0.043) *Mean (SD) Average overall HA: 2 w: 3.392 ± 2.106 (P) vs 4.220 ± 2.496 (C) (p < 0.018), 6 w: 3.678 ± 2.448 (P) vs 4.410 ± 2.544 (C) (p < 0.037) 2. Migraine frequency: NSD 3. HIT: NSD 4. GPE: NSD	PRF Group (n = 3): Worsened HA (n = 1); Swelling (n = 1); Rash (n = 1) Comparator: Dizziness (n = 8)); Swelling (n = 2); New onset eye (n = 2); pain and blurred vision (n = 1); Worsened HA (n = 2); Vomiting (n = 1)	Positive
Gabrhelik 2011;^32^ n = 30; P Pilot trial	Primary: *Pain intensity (VAS) Secondary: *MQS *GPE	3 m, 9 m	1. Median [IQR] VAS: *Baseline vs C* 3 m: 5.5 [4–7] vs 2.3 [0–6] p < 0.001 9 m: 5.5 [4–7] vs 4.3 [2–6] p < 0.05 *Baseline vs PRF* 3 m: 5.9 [4–8] vs 2.6 [0–5] p < 0.001 9 m: 5.9 [4–8] vs 3.1 [2–5] p < 0.001 2. Median [IQR] MQS – *Baseline vs C* 3 m: 9.2 [4.8–14.8] vs 4.8 [0–12.8] p < 0.001 9 m: 9.2 [4.8–14.8] vs 6.8 [0–14.8] p < 0.01 *Baseline vs PRF* 3 m: 9.2 [4.6–13.6] vs 3.2 [0–11.4] p < 0.001 9 m: 9.2 [4.6–13.6] vs 6.8 [0–11.4] p < 0.01	*Pain at injection site (n = 3)	Inconclusive
**NON-RANDOMIZED STUDIES**					
Alkaabi 2020,^33^ N = 30, P	Primary: *Pain intensity (NRS)	1 m, 6 m, 12 m	*NRS≤ 3/10 obtained for 27/30 at 1 m, 28/30 at 6 and 12 m *Baseline NRS unknown	NR	Positive
Batistaki 2021;^34^ N = 57, P	Primary: *HA frequency Secondary: *Pain intensity (NRS) *Analgesic consumption	1 m, 3 m, 6 m	1. Median (IQR) NRS: *Baseline vs PRF* Migraine cohort: - Baseline: 8.5 [8–10] - 1 m: 7 [5–8] p = 0.0005 - 3 m: 6 [5–8] p = 0.0001 - 6 m: 6 [5–8] p = 0.0001 Cluster cohort: - Baseline: 9 [8–9] - 1 m: NSD - 3 m: 8 [0–8] p = 0.0313 - 6 m: NSD ON/TTH cohort: - Baseline: 8 [7–9] - 1 m: NSD - 3 m: 4.5[4–8] p = 0.0156 - 6 m: NSD 2. HA frequency: *Baseline vs PRF* Migraine cohort: - Baseline: 14.5 [8–25] - 1 m: 2 [4–15] p = 0.0001 - 3 m: 6.5 [2–12] p = 0.0001 - 6 m: 6.5 [3–12] p = 0.0001 CH cohort: - Baseline: 15 [15–30] - 1 m: 2 [0–15] p = 0.0156 - 3 m: 2 [0–15] p = 0.0156 - 6 m: NSD ON/TTH cohort: - Baseline: 16.5 [15–30] - 1 m: NSD - 3 m: 4.5 [1–14] p = 0.0078 - 6 m: NSD	NR	Positive
Choi 2012;^37^ n = 10; R	Coprimary: *Pain intensity (VAS) *TPI Secondary: *Analgesic consumption	Monthly FU up to10 m.	Mean (SD) VAS: - Baseline: 6.9 (NR) - Post-PRF: 1.2 (NR) p < 0.001 - Last FU: 0.8 (NR) p < 0.001 . *Analgesic consumption: 8/10: Analgesic-free post-PRF 1/10: Substantial reduction 1/10: No change *TPI - Baseline: 232.7 - Post-PRF: 53.7 p < 0.001 - Last FU: 40.6 p < 0.001	None	Positive
Guner 2022;^38^ n = 25; R	Coprimary: *Pain intensity (VAS) *MIDAS *BPI *PSQI Secondary: *HA frequency *HA Duration	1 m, 3 m	1. Median (IQR)VAS - Baseline: 9 (8–10) - 1 m: 3(1–6) p < 0.001 - 3 m: 1(0–3) p < 0.001 2. Median (IQR) HA frequency (days/m): - Baseline: 10 (8–12) - 1 m: 5 (3–8) p < 0.001 - 3 m: 3 (0–5) p < 0.001 3. Median (IQR) HA episode duration (h): - Baseline: 48 (24–72) - 1 m: 12 (6–24) p < 0.001 - 3 m: 5 (3–12) p < 0.001 4. Median (IQR) PSQI: - Baseline: 16 (9–20) - 1 m: 9(3–15) p < 0.001 - 3 m: 5(2–10) p < 0.001 5. Median (IQR) MIDAS: - Baseline: 4 (3–4) - 1 m: 2(1–2) p < 0.001 - 3 m: 1(1–1) p < 0.001	NR	Positive
Huang 2012;^39^ n = 102; R	Primary: *Pain Relief Secondary: *Procedural Satisfaction	3 m	*≥ 50% pain relief: n = 52	*Temporary worsening of pain (n = 5)	Inconclusive
Karaoglan 2022;^43^ n = 27; R	Coprimary: *HA frequency *HA duration *Analgesic consumption *Pain intensity (VAS)	1 m, 3 m, 6 m	1. Mean (SD) Total HA frequency (episodes/m): - Baseline: 12.04 ± 2.83 - 1 m: 1.96 ± 4.56 p < 0.001 - 3 m: 2.48 ± 4.09 p < 0.001 - 6 m: 2.00 ± 1.00 p < 0.001 2. Mean (SD) Mild HA frequency (episodes/m): - Baseline: 2.48 ± 2.26 - 1 m: 0.81 ± 1.49 p = 0.001 - 3 m: 1.26 ± 1.23 p = 0.012 - 6 m: 1.00 ± 1.00 p = 0.015 3. Mean (SD) Moderate/Severe HA frequency (episodes/m): - Baseline: 9.44 ± 1.87 - 1 m: 1.22 ± 3.38 p < 0.001 - 3 m: 1.26 ± 3.19 p < 0.001 - 6 m: 1.00 ± 1.00 p < 0.001 4. Mean (SD) HA episode duration (h): - Baseline: 49.33 ± 19.78 - 1 m: 7.74 ± 20.72 p < 0.001 - 3 m: 9.93 ± 17.72 p < 0.001 - 6 m: 6.00 ± 6.00 p < 0.001 5. Mean (SD) Total triptan use (# tabs): - Baseline: 6.74 ± 1.75 - 1 m: 0.96 ± 2.85 p < 0.001 - 3 m: 1.00 ± 2.84 p < 0.001 - 6 m: 0.00 ± 1.00 p < 0.001 6. Mean (SD) Total analgesic use (# tabs): - Baseline: 11.00 ± 4.10 - 1 m: 2.48 ± 6.36 p < 0.001 - 3 m: 2.30 ± 3.81 p < 0.001 - 6 m: 2.00 ± 1.00 p < 0.001 7. Mean (SD) Highest VAS score: - Baseline: 8.89 ± 0.75 - 1 m: 1.44 ± 3.24 p < 0.001 - 3 m: 2.56 ± 3.24 p < 0.001 - 6 m: 4.00 ± 2.00 p < 0.001	None	Positive
Lee 2020;^40^ n = 45; R	Primary: *Proportion of pain reduction	1 m, 3 m, 6 m	1. Pain reduction: **>/ = 30%** - 1 m: n = 20 (44.4%) - 3 m: n = 23 (51.1%) - 6 m: n = 23 (51.1%) **>/ = 50%** - 1 m: n = 13 (28.9%) - 3 m: n = 17 (37.8%) - 6 m: n = 18 (40.0%) **>/ = 70%** - 1 m: 5 (11.1%) - 3 m: 9 (20.0%) - 6 m: 14 (31.1%)	None	Inconclusive
Li 2020;^35^ n = 20; P	Primary: *Pain intensity (VAS) Secondary: *BPI *Block efficacy	1 w, 1 m, 3 m, 6 m	1. Mean (SD) VAS: - Baseline: 7.1 - 1 w: 2.75 ± 0.27 p < 0.05 - 1 m: 2.25 ± 0.45 p < 0.05 - 3 m: 1.95 ± 0.47 p < 0.05 - 6 m: 1.75 ± 0.48 p < 0.05 2. Mean (SD) BPI: - Baseline: 45.05 ± 3.437 - 1 w: 10.06 ± 2.37 p < 0.05 - 1 m: 12.50 ± 2.46 p < 0.05 - 3 m: 12.90 ± 2.62 p < 0.05 - 6 m: 11.63 ± 2.98 p < 0.05 3. Block test efficacy obtained in 93.4% of cases (P < 0.0009).	*Transient cervicalgia (24 h) (n = 1) *Dizziness (30 min) (n = 3)	Positive
Li 2019;^41^ n = 139; R	Coprimary: *Izbicki Pain Score *QOL *NDI *Duration of relief	2y	1. Izbicki Pain Score at 2 yrs: 11.25 (P) vs 40 (C) (p < 0.001) 2. Median (IQR) HA frequency: 25 (0–75) (P) vs 50 (0–100) (C) (p < 0.001) 3. Median (IQR) Duration of relief: 8 m (P) vs 4 m (C) (p < 0.001) 4. *Mean (SD) Cognitive Function: 55.36 ± 19.82 (P) vs 46.82 ± 23.54 (C) (p = 0.026) *Mean (SD) Physical Function: 70.61 ± 29.47 (P) vs 47.87 ± 21.53 (C) (p < 0.001) 5. Mean (SD) Emotional Function: 61.17 ± 28.41 (P) vs 43.52 ± 25.48 (C) (p < 0.001) 6. Mean (SD) Sleep disturbance: 41.13 ± 14.36 (P) vs 78.22 ± 20.48 (C) (p < 0.001) 8. Mean (SD) Global health score: 59.31 ± 27.44 (P) vs 50.73 ± 21.90 (C) (p = 0.032)	None	Positive
Vanelderen 2010;^36^ n = 19; P	Primary: *Pain intensity (VAS) Secondary: *QOL *MQS	1 m, 2 m, 6 m	1. Mean (SD) VAS: - Baseline: 7.5 ± 0.4 - 1 m: 3.5 ± 0.8 p < 0.001 - 2 m: 3.5 ± 0.7 p < 0.001 - 6 m: 3.9 ± 0.8 p = 0.002 2.Likert (score of 6 or more): - 1 m: n = 13 (68.4%) - 2 m: n = 11 (57.9%) - 6 m: n = 10 (52.6%) 3. Mean (SD) Disturbance of daily activity: - Baseline: 6 ± 0.6 - 1 m: 3.2 ± 0.8 p = 0.035 - 2 m: 3.2 ± 0.8 p = 0.004 - 6 m: 3 ± 0.8 p = 0.02 4. Mean (SD) Mood disturbance: - Baseline: 4.8 ± 0.8 - 1 m: NSD - 2 m: 2.6 ± 0.8 p = 0.025 - 6 m: 2.7 ± 0.8 p = 0.037 4. Mean (SD) Sleep disturbance: - Baseline: 6.6 ± 0.8 - 1 m: 3.6 ± 0.9 p = 0.009 - 2 m: 3.5 ± 0.9 p = 0.009 - 6 m: 3.6 ± 1 p = 0.01 5. Mean (SD) MQS: - Baseline: 11.2 (R: 18.2) - 1 m: 4.4 (R: 18.2) p < 0.01 - 2 m: 3.4 (R: 17.1) p = 0.011 - 6 m: 2.2 (R: 17.1) p = 0.006	None	Positive
VanZundert 2003;^42^ n = 4; R	Primary: - GPE Secondary: - Duration of relief - Analgesic consumption	8 w- 2 yrs	1. GPE at 8 w: 4,7,2,7 2. Duration: no effect (n = 2), 18 m (n = 1), >24 m (n = 1) 3. Analgesic intake: reduced (n = 4)	None	Inconclusive
**CASE SERIES/CASE REPORTS**					
Belgrado 2021;^44^ n = 4; P	Primary: *HA frequency Secondary: *Duration of relief	1 w, 3 m	*Daily HA frequency: >50% reduction (n = 4); Complete response after 1 PRF (n = 2); complete response after 2 PRF (n = 1); complete response after 3 PRF (n = 1) *Duration of relief: 3.5 m (n = 1); 6 m (n = 1); 15 m (n = 2)	“Nearly all” patients reported mild local discomfort	Positive
Fadayomi 2019;^46^ n = 1; R	Primary: *Pain relief	2 w, 8 m	*Pain relief: 100% up to 8 w *all medications discontinued up to 8 w	None	Positive
Gonzalez 2020;^47^ n = 1; R	Coprimary: *Pain intensity *HA frequency Secondary: *Duration of Relief *Analgesic Consumption	NS	*Pain Intensity & Headache Frequency: - Post-First PRF: 80% reduction - Post-Second PRF: >90% reduction *Duration of effect: 12 m	None	Positive
Kwak 2018;^48^ n = 2; R	Coprimary: *Pain intensity *HA Frequency *HA Duration	2 w, 1 m, 2 m, 3 m	1. Mean (SD) NRS Patient 1: - Baseline: 8 (NR) - At 2 w-1 m-2 m-3 m:: 3 (NR) Patient 2: - Baseline: 7 (NR) - At 2 w, 1 m, 2 m, 3 m: 3 (NR) 2. HA frequency: NSD for both patients 3. HA duration: NSD for both patients	None	Positive
Lee 2015;^49^ n = 1; R	Coprimary: *Pain intensity *Duration of Relief	NS	1. Mean (SD) VAS Neck pain - Baseline: 6 (NR) - Last FU: 3 (NR) Mean (SD) VAS Occipital Pain - Baseline: 10 (X) - Last FU: 0 (X) 2. Duration: 6 m	None	Positive
Li 2018^50^; n = 1; R	Primary: *Pain intensity (VAS)	1 yr	1. Mean (SD) VAS: - Baseline: 8 (NR) - 1y: 0 (NR) 2. Return to work full-time.	None	Positive
VanderHoek 2013;^51^ n = 1; R	Primary: *Pain relief	NS	*NRS: 100% pain relief *Duration: Several months.	None	Positive
Voloshin 2021;^45^ n = 6; R	Coprimary: *Pain intensity (NRS) *HA frequency Secondary: *Duration of relief *Analgesic consumption	1 m, 2 m, 3 m, 4 m, 5 m, 6 m	Both NRS and HA frequency significantly improved up to 4 months	None	Positive
Zhang 2011;^52^ n = 2; R	Coprimary: *Pain intensity (NRS) *Duration of relief	NS	1. NRS Patient 1: 5/10 (baseline) to 0/10 (post-PRF) Patient 2: 4/10 (baseline) to 0/10 (post-PRF) 2. Duration of relief: 6 m	NR	Positive

BDI: Beck Depression Inventory; BPI: Brief Pain Inventory; ^o^C: Celsius; C: Comparator; CGRP: calcitonin gene-related peptide receptor; DA: distal approach; EORTC QLQ-30: European Organization for Research and Treatment of Cancer Quality of Life Questionnaire; ESI: epidural steroid injection; F: female; LMB: landmark-guided; FU: Follow up; GPE: global perceived effect; HA: headache; HIT: headache impact test; LA: local anaesthetic; M: male; m: month(s); MIDAS: Migraine Disability Assessment Scale; MQS: Medication Quantification Scale; N/A: not applicable; NDI: Neck Disability Index; NR: Not reported; NRS: Numeric Rating Scale; NS: Not specified; NSD: No statistical difference; P: Prospective study; PA: Proximal approach; PRF: Pulsed radiofrequency neuromodulation; PSQI: Pittsburgh Sleep Quality Index; QOL: quality of life; R: Range; RCT: randomized controlled trial; TPI: Total pain index; USG: ultrasound-guided; V: volt; VAS: Visual Analog Scale; W: Watt; w: weeks; y: years.

**Table 3.** Cochrane risk of bias tool for RCTs 2.0

Author, year	Domain 1	Domain 2		Domain 3	Domain 4	Domain 5	Overall Risk of Bias
	Randomization Process	Assignment to Intervention	Adherence to Intervention	Missing Outcome Data	Measurement of Outcome	Outcome Reporting	
Cohen^31^, 2015	+	+	+	±	+	+	±
Gabrhelik^32^, 2011	±	+	+	±	+	+	±

+ = low risk, – = high risk, ± = some concerns.

**Table 4.** The Risk of bias in non-randomized studies of interventions (ROBINS-I) assessment tool

Author, year	Confounding		Participant Selection		Classification of Interventions		Deviations from Intended Interventions		Missing Data		Outcome Measures		Outcome Reporting		Overall Risk of Bias	
	RoB	Direction	RoB	Direction	RoB	Direction	RoB	Direction	RoB	Direction	RoB	Direction	RoB	Direction	RoB	Direction
First author, year																
Alkaabi^33^, 2020	--		--		+		+		---	AN	---	AN	--	AN	---	AN
Batistaki^34^, 2021	+		+		+		+		+		-	AN	+	AN	-	
Choi^37^,2012	-		+		+		+		-		-	AN	-	AN	-	AN
Guner^38^, 2022	-		+		+		+		+		+		-		-	
Huang^39^, 2012	--		-		+		+		+		--		--	TN	--	TN
Karaoglan^43^, 2022	-		+		+		+		-		-	AN	-	AN	-	AN
Lee^40^,2020	-		-		+		+		-		-		-		-	TN
Li^41^,2019	--		--		-		+		-		-	AN	--	AN	--	AN
Li^35^,2020	-		-		+		+		+		-	AN	-	AN	-	AN
Vanelderen^36^,2010	+		+		+		+		+		-	AN	-	AN	-	
Van Zundert^42^, 2003	-		-		+		+		-		-		--		--	

+ = low risk, - = moderate risk, -- = serious risk, --- = critical risk,

TN = toward null, AN = away from null.

**Table 5.** Quality appraisal checklist for case series studies.

Author, year	1	2	3	4	5	6	7	8	9	10	11	12	13	14	15	16	17	18	19	20
Belgrado^44^, 2021	+	-	-	-	+	+	-	+	+	±	-	±	+	±	+	+	-	+	+	±
Voloshin^45^,2021	+	±	±	±	+	+	-	+	+	+	-	+	+	+	+	+	+	+	+	+

+ = yes, – = no, ± = partial/unclear.

One study compared cervical epidural steroid injections (CESI) (n = 52) to CESI combined with PRFN (n = 87).^41^ Significant long-term reduction in headache intensity was demonstrated in both groups, with the group combining CESI with PRFN (42°C, 60 Hz, 300s) to be found superior. Notably, in this study, 8 patients who failed to obtain relief from CESI were successfully treated with PRFN afterward. Five refractory patients from the PRFN + CESI group underwent a second PRFN treatment, with three patients experiencing subsequent relief. Median duration of pain relief was 8 months (CESI+PRFN cohort) versus 4 months (CESI group). In terms of secondary outcome measures, overall quality of life with respect to cognitive function (p = 0.026), emotional functioning (p < 0.001) and physical function (p < 0.001) was improved in the PRFN group versus comparator. Sleep quality was also significantly better in the PRFN group (p < 0.001).

Another retrospective cohort study assessed the analgesic effect of three PRFN cycles (42°C, 90s).^40^ At 6 months post-intervention, 51% of patients demonstrated to have a 30% or more pain reduction from baseline.

In a third study, only 2 out of 4 patients receiving PRFN (42°C, 45 V, 20 ms PW, 120s) experienced successful pain reduction, lasting 18 and 24 months, respectively.^42^ One patient experienced no pain reduction and one patient experience a worsening of pain at the 8-week follow-up timepoint. Analgesic intake was reported to be reduced in all four patients.

Another prospective cohort study involved 20 patients, of which two presented with CEH.^35^ They were treated with a single cycle of USG PRFN (42°C, 65 V, 2 Hz, 20 ms PW, 900sec). Overall, there were significant decreases in both headache intensity and BPI scores at 6 months post-intervention (mean headache intensity from 7.1 to 1.75, p < 0.05).

We found one case report of two patients who were treated with XRG PRFN (42°C, 240 sec).^52^ Baseline NRS pain intensities were moderate (4/10 and 5/10). Following treatment, both patients had complete resolution of pain lasting 6 months.

#### CM

In one case report, a female with CM was completely headache free up to one year, after a combined USG and CTG single-cycle PRF (42°C, 45 V, 2 Hz, 20 ms PW, 900 sec). The patient was able to return to work full-time with headache intensity (VAS) reduced from a baseline of 8/10 to 0/10 at 1-year post-treatment.^50^

#### CH

In one case report, a male with chronic CH was treated with one session of PRFN (42°C, 90s) and reported complete pain relief of retrobulbar pain up to 8 months post-treatment. The patient was able to discontinue all analgesics.^46^

#### Other

We found one case report investigating PRFN at C2 level for headache and neck pain due to atlanto-axial subluxation (AAS).^49^ Baseline pain scores were high and refractory. After three cycles of PRFN (42°C, 120 sec), her neck pain intensity significantly decreased and occipital pain completely resolved at 6 months.

### Distal GON Approach

We found 11 studies (two RCTs,^31,32^ six cohort studies,^33,34,36,37,39,43^ one case series,^44^ two case reports^47,51^) assessing the effect of PRFN treatment targeting the distal GON. Eight studies used LMG technique,^31–34,36,39,43^ one study used XRG,^37^ and one case report USG.^51^ The remaining two studies did not specify any use of image guidance technique.^44,47^

#### Summary of Results with Distal Approach

A total of 362 patients were analyzed in the 11 studies that utilized the distal approach to PRFN of the GON.^31–34,36,37,39,43,44,47,51^

For CEH, significant reductions in headache intensity and analgesic consumption were observed at 3 and 9 months in one pilot trial.^32^ However, these findings in CEH should be noted with caution due to the study’s design as a pilot trial with a smaller sample size. For CM, two studies^34,43^ (n = 65) reliably demonstrated significant relief in terms of pain relief up to 6 months^34,43^ and reduction in MHD^34,43^ up to 6 months, and reduction in analgesic intake.^43^ For CH, the current evidence is inconclusive. For ON, one RCT^31^ comparing PRFN to LA/steroid with sham PRFN and four out of five observational studies^34,36,37,39,51^ (n = 82) demonstrated significant pain relief from 6,^34,36,51^ to 10 months. We found one case report for the indication of SUNCT, with significant decrease in terms of headache frequency and headache intensity, up to 12 months.^47^

#### CEH

We found one pilot RCT applying PRFN to the distal part of GON for CEH disorder.^32^ Patients were randomly allocated to single LMG PRFN treatment (42°C, 45 V, two cycles of 120 seconds) or GONB with LA and steroids. Authors concluded that both groups achieved significant reduction in headache intensity and analgesic consumption at 3 and 9 months, however these results need to be assessed under scrutiny due to the type of study (pilot trial).

#### CM

Two prospective cohort studies^33,34^ and one retrospective cohort study^43^ employed the distal GON approach for PRFN in the management of CM. In the first prospective study, 30 patients received a LMG single PRFN treatment (42°C, 42 V, 180 seconds, 3 cycles).^33^ NRS of 3/10 or less was achieved in 27 patients (90%) at 1 month, and in 28 patients (93%) at 6 and 12 months. However, the study was deemed to have a critical risk of bias due to confounding, missing data and lack of adequate outcome reporting.

In the second study, 57 patients received one LMG GON PRFN treatment (42°C, 40–60 V, 360 sec).^34^ Thirty-eight patients within the cohort had a diagnosis of CM with an average baseline NRS of 8.45 (SD 1.28) and median 14.5 [interquartile range; IQR 8–25] headache days per month. Monthly headache days (MHD) was significantly reduced from baseline (14.5 [8–25] MHD) at one month (2 [4–15] MH; p = 0.0001), at 3 months (6.5 [2–12]; p = 0.0001), and at 6 months (6.5 [3–12]; p = 0.0001). Headache intensity was also significantly reduced from baseline (8.5 [8–10]) to 1 month (7 [5–8]; p = 0.0005), at 3 months (6 [5–8]; p = 0.0001) and at 6 months (6 [5–8]; p = 0.0001). There was no significant change in analgesic consumption.

In one retrospective cohort study, 27 migraine patients received a single PRFN treatment one month after inadequate response to GONB (42°C, 45 V, 360 sec at 5 Hz, 5 mm PW).^43^ All patients had significant improvement post-PRFN as compared to baseline, up to 6 months, in terms of MHF (post-PRFN 2.00 ± 1.00 vs baseline 12.04 ± 2.83; p < 0.001), headache episode duration in hours (post-PRFN 6.00 ± 6.00 vs baseline 49.33 ± 19.78; p < 0.001), and total analgesic consumption (post-PRFN 2.00 ± 1.00 vs baseline 11.00 ± 4.10; p < 0.001).

#### CH

One prospective cohort study reported a significant difference in headache intensity at 3 months (NRS 9 [IQR 8–9] vs 8 [IQR 0–8]; p = 0.0313) but not at 6 months.^34^ The MHD was significantly reduced from baseline (15 [IQR 15–30]) to 1 month (2 [0–15]; p = 0.0156) and at 3 months (2 [0–15]; p = 0.0156) but not at 6 months. No significant difference in overall analgesic consumption was reported.

In a second study, two out of four patients reported complete relief of CH up to 6 and 15 months, respectively, after one PRFN treatment.^44^ For the remaining patients, PRFN treatment was repeated with subsequent complete relief up to 15 months.

#### ON

A total of six studies including one RCT and five observational studies evaluated the effects of GON PRFN in patients with a diagnosis of ON.^31,34,36,37,39,51^

We found one RCT comparing PRFN (42°C, 40–60 V, 20 msec PW, 3 × 120 sec) to GONB with LA and steroid followed by sham PRFN.^31^ The PRFN group demonstrated significant and superior pain reduction up to 6 months post-intervention as compared to baseline. (mean change from baseline for the PRFN group versus steroid group: –1.413 ± 2.352 vs –0.33 ± 1.382; p = 0.017). No significant differences were noted between groups in terms of headache frequency, depression, rescue analgesic consumption, or quality of life.

A small retrospective study on 10 patients investigated the effect of single XRG PRFN (42°C, 240 sec/cycle, duration unspecified).^37^ Mean pain scores were significantly improved from baseline to last follow-up at 10 months post-intervention (6.9 vs 0.8; p < 0.001). Analgesic consumption was significantly decreased.

A large retrospective observational study of 102 patients assessed the effect of LMG GON PRFN (42°C, 40–60 V, 150–500 Ω, 120 seconds/cycle).^39^ The study was deemed inconclusive, as only 52 patients (52%) experienced greater than or equal to 50% pain relief and procedural satisfaction with GON PRFN treatment lasting a minimum of three months.

Another prospective cohort study investigated the effect of a single LMG PRFN (42°C, 45 V, 240 sec/cycle).^36^ VAS headache intensity significantly reduced from baseline (mean 7.5 ± 0.4) to 1 month (3.5 ± 0.8; p < 0.001), at 2 months (3.5 ± 0.7; p < 0.001), and at 6 months (3.9 ± 0.8; p = 0.002). Disturbance of daily activity, mood and sleep disturbance and analgesic consumption each significantly improved at 1 month, 2 months, and 6 months follow-up, as compared to baseline.

Lastly, VanderHoek et al. described a case report of a 32-year-old male patient who received a single treatment of USG GON PRFN (38–42°C; 2 × 120 sec).^51^ The patient experienced 100% pain relief for several months.^51^

#### SUNCT

Only one study assessed PRFN of the distal GON in a 75-year-old female with longstanding history of SUNCT.^47^ She had previously been treated with three GONB with LA and steroid with a positive response lasting 3–4 months but this effect started to wane. She was then treated with two PRFN treatments (42°C; 2 × 120 sec), each treatment 8 months apart, each treatment giving her 80% reduction in headache frequency and headache intensity, up to 12 months.

### Proximal GON Approach

We only found three studies (one retrospective cohort, one case series, one case report)^38,45,48^ utilizing a proximal PRFN approach, all performed with USG in patients with CM (n = 33).

#### Summary of Results with Proximal Approach

A total of 33 patients – solely CM patients – across three studies were analyzed using the proximal approach to PRFN of the GON.^38,45,48^ All studies reported a substantial reduction in headache intensity and frequency from 3 months^38,48^ to 6 months.^45^

#### CM

In a first retrospective cohort study (n = 25), patients received a single PRFN cycle of 360-second duration.^38^ Headache intensity, MHD and average headache duration were significantly improved up to 3 months’ post-intervention. Significant improvements in sleep quality (as measured with the Pittsburgh Sleep Quality Index (PSQI)) and overall migraine disability on patient life (as measured with the Migraine Disability Assessment (MIDAS)) were also observed at both the one and three month follow up timepoints.

A small case series (n = 6) looked at the effect of a single 360 second cycle of USG PRFN.^45^ NRS headache intensity and headache frequency decreased significantly from baseline up to 6 months (p < 0.05), with two patients remaining completely free of pain at 6 months post-PRFN. Three out of six patients were reported to have halved their daily analgesic consumption, and they began to appreciate again a positive effect from their abortive therapy (triptans and/or NSAIDs).

One case report on two patients with severe CM reported favorable outcomes in terms of headache intensity in both patients up to 3 months, but no significant change in headache duration or frequency.^48^

### Adverse Effects and Complications of PRFN Treatment

Only five studies reported on the adverse effects of PRFN treatment.^31,32,35,39,44^ Four out of five studies employed the distal approach.^31,32,39,44^ Overall, only 3.1% of all study patients included in this review reported mild and temporary adverse effects that included worsened headache (<10 days), cervicalgia, local discomfort, dizziness, rash and localized swelling, pain at injection site lasting >24 hours, pain exacerbation or new onset retro-auricular pain, which subsided within 3 weeks. No serious complications were reported on the 3 PRFN approaches across all studies.

## Discussion

This is the first systematic review analyzing the therapeutic role of PRFN of the GON in the treatment of headache disorders. Our extensive literature search found 22 studies of which only two RCTs, with the overall quality of evidence rated of low quality. The present data indicates an analgesic benefit from PRFN treatment for ON and CM with occipital tenderness. Studies demonstrated PRFN to provide significant analgesia and reduction of MHF in CM from 3 to 6 months; and significant pain relief for ON from 6 to 10 months. Mild adverse effects were reported in 3.1% of cohort. The analgesic benefit of PRFN for other headache disorders remains unclear at this time. There is no clear superiority of certain PRFN settings, anatomical approach or image guidance identified demonstrated in this review.

### PRFN Impact on Headache Pain and Pain-Associated Domains

Current evidence indicates there is some evidence for analgesic benefit from PRFN for GON for ON and migraine with occipital tenderness. However, the analgesic benefit of PRFN for other headache disorders is unclear due to low number and quality of included studies and needs to be further investigated.

Most of the studies did not consequently investigate nor report impact of PRFN on pain-associated domains. Only eight studies reported impact on analgesic consumption, seven studies demonstrated a significant decrease ^32,36,37,42,43,45,47^ Furthermore, six out of only eight studies^34,38,43–45,47^ reporting on MHF showed a significant decrease, of note the only RCT investigating this secondary outcome reported no significant decrease between groups. There is a lack of use of validated questionnaires to measure these secondary outcomes across all studies.

### Impact of Choice of Target

Although the first impression of the data might suggest that PRFN of GON may be effective for all headache types and across all targets, new insights are gained when closer reviewing the data. As for the ON patient population, we only found studies targeting the GON at the distal approach. Therefore, no statements can be made as to the efficacy of PRNF on the other targets for this population. As for CM, there is currently only one case report to support the use of PRFN at the C2 level. The majority of evidence focuses and supports the use of distal and proximal GON approach, but no statement can be made as to target superiority due to lack of comparative study. We did find a previous study comparing the efficacy of distal versus proximal GONB for CM that demonstrated no significant difference in efficacy.^12^

As for the CEH population, the majority of studies investigate and support the choice of C2 target for PRFN. Interestingly, we only found one study (RCT) concluding both PRFN and GONB with LA and steroid delivered significant pain relief when applied at the distal GONB approach.^32^ Although not superior, this study indicates that PRFN could be applied with long-term effect in CEH patients when steroids are contra-indicated. We did not find any studies investigating the use of proximal PRFN for CEH.

### Impact of Image Guidance

When comparing image guidance techniques in the included studies, we did not notice a substantial difference in efficacy. Our review only discovered three studies^39,40,42^ that reported a negative outcome of PRF of GON for headaches, one study using LMG,^39^ one study using XRG,^40^ and one study did not specify approach.^42^ This small number makes it impossible to draw any conclusion about the potential correlation between efficacy and image guidance techniques.

### The Use of PRFN versus GONB with LA/steroid

GONB has been shown to be a cost-effective, minimally invasive, and safe intervention for headache management.^53^ However, (chronic) steroid exposure is associated with a wide range of harmful effects.^54^ PRFN has been shown to be an easy-to-use interventional treatment associated with minimal tissue trauma without the risks associated with steroid administration.^55^ The application of PRFN for other pain indications has been demonstrated to provide long-lasting relief, similar or even longer than the duration of LA and steroid.^26,56^ Our evidence also suggests that PRFN is associated with longer duration of relief versus GONB with LA/steroid for the indication of headaches. Thus, PRFN presents as a potentially more effective and safe treatment alternative for the headache patients, especially for those chronic headache patients refractory to standard treatment who are likely to return for repeated procedures.

### Relation to Other Studies

Few systematic reviews have been published exploring the effectiveness of both PRFN and radiofrequency ablation (RFA) in the treatment of headache disorders and facial pain.^57–59^ Two systematic reviews explored the efficacy of both PRFN and RFA to the zygapophysial joints of the cervical spine in the treatment of cervicogenic headaches only.^58,59^ The third systematic review explored the available evidence on both PRFN and RFA for a range of different anatomical targets (C1C2 joint, GON, suprascapular nerve) for headaches.^59^ This review however only included literature published up to 2019 and the authors only included three studies on the use of PRFN for GON. Further, when presenting the evidence of PRFN for GON for headaches, the authors did not stratify the data according to anatomical GON target.

Our systematic review is the only systematic review focused solely on PRFN of the GON in the treatment of all headache types. Our review is unique in its organization and reporting of studies based on the three anatomical approaches targeting the GON, while giving a comprehensive overview of the evidence for each different headache type.

### Safety Profile

PRFN has been widely accepted as a safe and effective analgesic treatment for different chronic pain syndromes.^60^ The incidence of side-effects and complications in the studies reported in this review was low and most side-effects were mild and resolved spontaneously. Our review did not indicate any overt differences in safety across different PRFN settings. However, we did note that of the five studies that reported adverse effects, four studies had used a LMG approach when targeting the distal GON,^31,32,39,44^ advocating for image-guidance when performing nerve blocks. This is in concordance with previous studies in interventional pain practice, indicating that use of image-guidance improves safety and accuracy.^61^ USG has been advocated as the safest option compared to CTG or XRG, due to the lack of radiation.^61^

### Limitations

This review has several limitations. Despite the potential for benefit of PRFN in the treatment of headaches, only a few high quality prospective RCTs have evaluated this question. The majority of the included data comes from observational studies, with inherent risk of bias and confounding. The quality of the included studies was moderate to low. Further, the majority of those studies did not include a control group. The heterogeneity across studies was also significant, with large differences in terms of headache indication, intervention parameters (e.g., anatomical PRFN target, PRFN settings, duration of treatment) image guidance technique, and comparators that made interstudy comparison challenging.

### Future Research

Future comparative studies should therefore focus on determining the best anatomical target, optimal intervention parameters, and image-guidance technique for PRFN treatment for each headache disorder. Although the observational studies suggest a beneficial effect of PRFN for CM, high-quality comparative trials are needed to fully assess and establish the efficacy of PRFN as a treatment for headaches. Future trials could also benefit from focusing on secondary health outcome measurements in different headache populations, such as adding MHF, QOL, sleep, and validated headache questionnaires, such as HIT-6^62^ or MIDAS^63^ to explore the full effects of PRFN treatment.

## Conclusions

Low-quality evidence indicates an analgesic benefit from PRFN for GON for ON and migraine with occipital tenderness but more evidence is needed to clearly confirm that it is substantially better than a blockade with LA and steroids. The role of PRFN for other headache disorders needs to be more investigated. Due to considerable heterogeneity across studies and lack of comparative studies, it remains difficult to formulate clear recommendations as to optimal PRFN target, settings, and location. High-quality RCTs with allocation concealment and blinding of outcome assessors and participants are required to further explore the role of this intervention.

## Acronym List

AAS: Atlantoaxial subluxation; CEH: Cervicogenic headache; CESI: Cervical epidural steroid injection; CH: Cluster headache; CM: Chronic migraine; CTG: Computed tomography guidance; DRG: Dorsal root ganglion; GON: Greater occipital nerve; GONB: Greater occipital nerve block(s); HIT-6: Headache impact test; ICHD: International classification of headache disorders; LA: Local anesthetic(s); LMG: Landmark-guided; MHD: Monthly headache days; MHF: Monthly headache frequency; MIDAS: Migraine disability assessment; NRS: Numeric rating scale; OCIM: Obliquus capitis inferior muscle; PRISMA: Preferred reporting items for systematic reviews and meta-analysis; PRFN: Pulsed radiofrequency neuromodulation; RCT: Randomized controlled trial; RFA: Radiofrequency ablation; SR: Systematic review; SSCM: Semispinalis capitis muscle; SUNCT: Short-lasting unilateral neuralgiform headache attacks with conjunctival injection and tearing; TAC: Trigeminal autonomic cephalalgia; TCC: Trigeminocervical complex; TTH: Tension type headache; USG: Ultrasound-guided; VAS: Visual analog scale; XRG: X-ray guided.

## Attestation

Kyle De Oliveira participated in the conception of the review, acquisition of data, analysis, interpretation of data, and drafting of the manuscript.

Nina D’hondt participated in the acquisition of data, analysis, and interpretation of data and review of manuscript.

Marina Englesakis participated in the execution of database search strategies, management of database results, and review of the manuscript.

Akash Goel participated in the interpretation of data and review of the manuscript.

Yasmine Hoydonckx participated in the conception of the review, acquisition, analysis, interpretation of data, and drafting the manuscript.
